# WHY CULTURAL CONTEXT IS VITAL TO STUDY LONGEVITY IN JAPAN

**DOI:** 10.1093/geroni/igae098.0383

**Published:** 2024-12-31

**Authors:** Yasuyuki Gondo

**Affiliations:** Osaka University, Suita, Osaka, Japan

## Abstract

Due to the worldwide expansion of the aging population, the number of centenarians has been increasing over the last decade. However, the centenarian population ratio is highly diverse among countries. The cause of diversity tends to be attributed to differences in the country’s economic situation. However, cultural differences in attitudes toward aging and deference to older people are potentially influential factors. In the case of Japan, regardless of its higher centenarian ratio in the world, there is a larger bed-bound population of centenarians. This unique situation is caused by combining a universal insurance system supported by a strong economy, a traditional healthy diet, and motivation to maintain good health. However, rather than those, a conventional attitude of deference to older people would be an essential cause of longevity in Japan. In Japan, Respect for the Aged Day is a national holiday. It was enacted in 1963 based on the Act on Social Welfare for the Elderly. With this timing, many local governments start presenting cash to older people. Currently, these cultural-based policies seem to have reached a turning point. A multi-country survey reported that only 25% of Japanese people wish to live to 100, the lowest among countries. At the same time, the main reason not to wish for a long life was “dislike to be a burden to people around” by observing a more significant number of bed-bound older people daily. This negative motivation might accelerate healthy behavior and cause further longer life, ironically.

